# A Real-Time Intelligent Valve Monitoring Approach through Cameras Based on Computer Vision Methods

**DOI:** 10.3390/s24165337

**Published:** 2024-08-18

**Authors:** Zihui Zhang, Qiyuan Zhou, Heping Jin, Qian Li, Yiyang Dai

**Affiliations:** 1 School of Chemical Engineering, Sichuan University, Chengdu 610065, China; 2021141490096@stu.scu.edu.cn (Z.Z.); 2021141490094@stu.scu.edu.cn (Q.Z.); 2 China Three Gorges Corporation, Beijing 100038, China; jinheping@ctg.com.cn (H.J.); li_qian10@ctg.com.cn (Q.L.)

**Keywords:** valve monitoring, computer vision, loss prevention, regional convolutional neural network, feature pyramid network, coord attention

## Abstract

Abnormal valve positions can lead to fluctuations in the process industry, potentially triggering serious accidents. For processes that frequently require operational switching, such as green chemical processes based on renewable energy or biotechnological fermentation processes, this issue becomes even more severe. Despite this risk, many plants still rely on manual inspections to check valve status. The widespread use of cameras in large plants now makes it feasible to monitor valve positions through computer vision technology. This paper proposes a novel real-time valve monitoring approach based on computer vision to detect abnormalities in valve positions. Utilizing an improved network architecture based on YOLO V8, the method performs valve detection and feature recognition. To address the challenge of small, relatively fixed-position valves in the images, a coord attention module is introduced, embedding position information into the feature channels and enhancing the accuracy of valve rotation feature extraction. The valve position is then calculated using a rotation algorithm with the valve’s center point and bounding box coordinates, triggering an alarm for valves that exceed a pre-set threshold. The accuracy and generalization ability of the proposed approach are evaluated through experiments on three different types of valves in two industrial scenarios. The results demonstrate that the method meets the accuracy and robustness standards required for real-time valve monitoring in industrial applications.

## 1. Introduction

In the process industry, valves play a pivotal role in process control, cut-off, regulation, diversion, countercurrent prevention, pressure stabilization, and other functions. In the event of valve failure, negative consequences can arise, including asset loss, production loss due to plant shutdowns, and health, safety, and environmental (HSE) issues [1,2]. For instance, in 1997, a major fire occurred at the Beijing Dongfang Chemical Plant due to workers incorrectly operating valves while unloading light diesel oil, resulting in 9 fatalities, 39 injuries, and direct economic losses amounting to CNY 117 million [3]. Similarly, in 2020, a major explosion at the Dahej Chemical Plant in India occurred due to improper handling of a valve, leading to the release of hazardous chemicals, resulting in 10 fatalities and several injuries [4]. These incidents underscore the paramount importance of mechanical integrity in process safety management, particularly in valve monitoring. In recent years, as countries have begun to pursue low-carbon and green development, the use of renewable energy for hydrogen production, followed by the synthesis of green ammonia, green methanol, and other chemical products, has garnered significant attention. However, due to the volatility of renewable energy sources, the production processes of green ammonia and similar facilities frequently require adjustments in production load. Consequently, the operation of valves becomes more frequent. Therefore, valve monitoring is of paramount importance in chemical production processes based on renewable energy.

In industrial applications, some critical valves are already equipped with detection functions, and there is a body of academic research focused on valve monitoring. However, these studies often focus on control valves [5,6,7], pressure relief valves [8,9], or valve leakage detection [10,11].

Due to the extensive presence of valves throughout the plants of process industry and the limited scope and frequency of routine inspections, timely detection of abnormalities in these valves is often challenging [12,13]. Additionally, the large number of valves and their varying positions in normal conditions make it difficult for inspectors to quickly identify any abnormal changes in valve positions. Although many plants have hundreds of video cameras installed, with most important manual valves within their monitoring range, control rooms can only display a limited number of camera feeds. Human operators cannot simultaneously monitor the status of each valve. Consequently, if a valve experiences an abnormal position due to equipment failure or human error, it is difficult to detect promptly. During this period, the abnormal valve position may affect the normal operation of the equipment. Only when other process variables trigger an alarm do operators use comprehensive judgment to troubleshoot and locate the abnormal valve, which can be time-consuming. This significantly increases maintenance costs, resulting in direct economic losses. If not detected in time, these issues could also lead to serious incidents.

Given these challenges, it is essential to devise an automated method for monitoring abnormal valve positions. Traditional valve position monitoring typically requires significant costs to purchase sensors for each valve and aggregate all sensor signals into a central control system [14,15]. This greatly increases system computation and cost. Over the past decades, the growth of artificial intelligence (AI) has allowed industries to automate and improve their operational efficiency [16,17]. With advancements in computational power and reductions in imaging sensor costs, the use of computer vision methods for hazard detection through ubiquitous cameras in the process industry has become quite mature [18]. Therefore, real-time valve position detection based on cameras has become possible.

Since AlexNet [19] won the ImageNet Large Scale Visual Recognition Challenge (ILSVRC) with its deep convolutional neural network (CNN) model, CNNs have revolutionized computer vision and pattern recognition. Deep CNNs can extract features and classify objects within a single network, learning comprehensive object features to achieve better detection performance. They have been widely used in the fields of process safety monitoring such as fire detection [20,21,22], smoke detection [23,24,25], gas leak detection [26,27,28], and safety helmet detection [29,30,31].

Currently, there have been some reports on computer vision-based valve monitoring research. Li et al. [32] proposed a novel solution with a specific measurement system of the valve opening area that enhances the visibility of valve openings by combining top lighting and camera exposure. A partial image is used to extract the single closed edge of the valve opening. The area computation based on the minimum circumscribed circle and the maximum inner circle of the edge can then be used to identify the valve opening status. However, in practical applications, it is difficult for cameras to capture images of the valve opening. Ahmed et al. [33] proposed an instrumentation valve (IV) status monitoring system based on optical camera communication (OCC). A transmitter circuit with a temperature sensor is integrated into each IV, and a closed-circuit television (CCTV) camera is used to receive data. The valve angle is measured in a cloud server using the data received by the CCTV to determine angle changes due to the proper closing or opening of the instrument valve. Xu et al. [34] proposed a cone valve seal detection method based on the YOLOv3 framework to address the limitations of traditional manual inspection, achieving 95.5% accuracy and a detection speed of 15 images per second. This automated approach enhances efficiency and provides reliable supervision for seal test qualification during cone valve inspection.

Currently, there has been little research on using existing industrial cameras for valve position monitoring. However, some researchers have used computer vision-based methods for knob gear recognition, which is similar to valve position detection. Qin et al. [35] proposed a three-stage knob gear recognition method for substations, utilizing YOLOv4 and Darknet53-DUC-DSNT models to address challenges such as low signal-to-noise ratio, image deformation due to shooting angles, and inconsistent feature distribution. YOLOv4 is employed as the knob area detector, while Darknet53, combined with the DUC and DSNT structures, enhances feature extraction and spatial generalization. This method calculates knob gear angles by analyzing the line from the rotating center point to the pointing point, significantly improving the performance and accuracy of knob gear detection in unattended substations.

Sun et al. [36] proposed a detection model for high-voltage cabinet switch clusters. They utilized EfficientNet-B0 as the backbone network, which is well suited for multi-scale target detection. To enhance the accuracy of small target extraction, they designed a multi-scale feature fusion neck network. This neck network incorporates the concept of the bidirectional cascade (BiC) module from the RepBi-PAN network, omitting the re-parameterization module to improve the real-time performance of the valve switch feature fusion module. Additionally, they developed a valve switch edge capture structure to enhance switch detection accuracy. This method significantly improves the efficiency and accuracy of switch status detection in numerous high-voltage cabinets in substations.

Furthermore, researchers in the field of wind power generation have also employed computer vision methods to identify the rotor blades of wind turbines, which share similarities with the rotational motion trajectories of valves in the chemical industry. Wu et al. [37] developed the YOLOv8-ScConv-WIoU model by incorporating space and channel reconstruction convolution (ScConv) and Wise-IoU (WIoU) loss functions. They demonstrated their model on a self-constructed dataset containing coded labels of different rotation positions and paste positions of the fan rotor. Their method achieved faster convergence, achieved significantly improved accuracy and robustness, and provided new perspectives and insights for the application of other object detection tasks in the industrial field.

In this paper, a real-time valve position monitoring approach based on a computer vision method is proposed. This approach captures minor changes in valve positions in real time identifies them. Its aim is to achieve precise monitoring of valve position changes, thus maximizing the avoidance of potential dangers and accidents. Region-based convolutional neural network (RCNN) is introduced for extracting valve features based on YOLOv8, and a feature pyramid network (FPN) is utilized for feature fusion. Additionally, a coordinate attention (CA) mechanism, tailored to the characteristics of production scenes, is incorporated to embed position information into feature channels for the more accurate extraction of valve rotation features. Finally, a rotation algorithm is employed to calculate the valve position using the coordinates of the valve’s bounding box.

## 2. Methods

### 2.1. Framework

**Figure 1:**
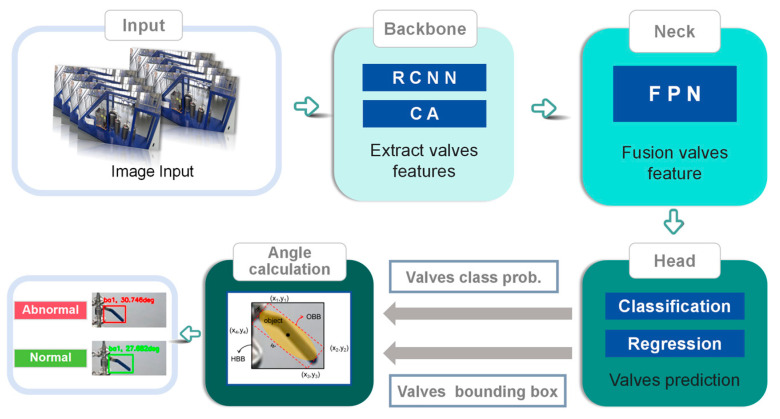
Framework of the real-time intelligent valve monitoring approach.

The proposed intelligent valve monitoring approach is depicted in Figure 1, consisting of three primary steps. Initially, images of valves and their surrounding environments are captured using industrial-grade cameras, forming a dataset for the YOLO model utilized in valve monitoring. Subsequently, real-time image data are processed frame by frame through the YOLO model, facilitating the extraction of edges, corners, and fundamental texture features of the valves. Finally, the valve position is determined by calculating the rotation angle of the valve based on the extracted features. If the calculated angle exceeds a predefined threshold, indicating an anomaly, an alarm is triggered.

### 2.2. Dataset Process

**Figure 2:**
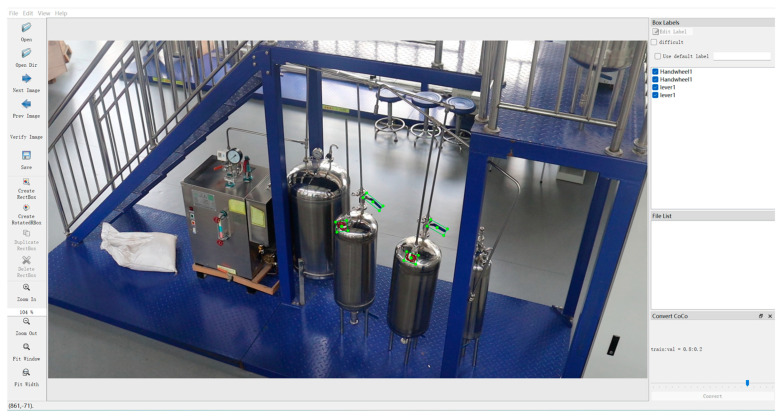
Valves labeling process.

For the collected valve images in industrial scenarios, the initial step involves annotating and classifying the valves. This annotation process, as demonstrated in Figure 2, entails providing the model with the boundary box marked with the green box in the figure, inclusive of the coordinates and angles of the four corners, as well as valve category information. To ensure standardized and consistent angle information, valves of the same category are rotated clockwise based on the horizontal direction to align with the valve rotation angle. These details are critical for the angle calculation results and subsequent model input. For partially obscured or circular-shaped valves, specific markings are created beforehand to facilitate the effective extraction of valve position characteristics, thereby aiding in more accurate calculations of the valve’s rotation degree. Further details on specific markings are provided in Section 3.2. Additionally, although the training data were acquired under optimal lighting conditions, brightness adjustment preprocessing was applied to some images prior to training. This approach enhances the model’s adaptability to varying lighting conditions.

### 2.3. Valve Feature Extraction by YOLO V8

**Figure 3:**
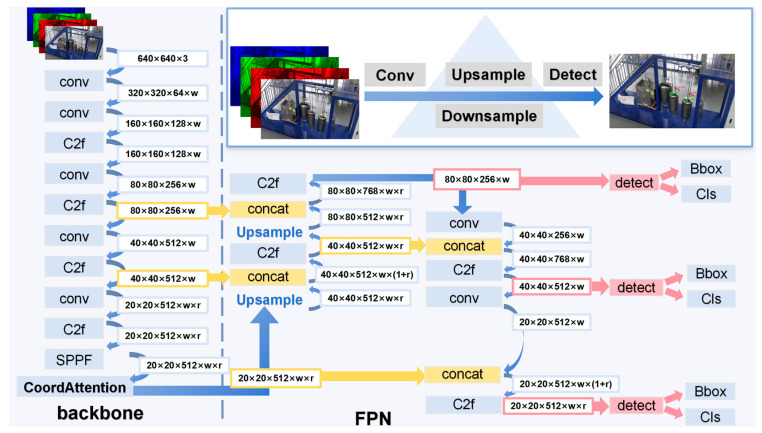
Network structure based on YOLOv8 for valve feature extraction.

The valve feature extraction model proposed in this paper is based on structural adjustments from YOLO V8, as illustrated in Figure 3.

It includes feature extraction based on regional convolutional neural networks (RCNNs), small target feature enhancement using the coord attention (CA) mechanism, and multi-scale feature fusion employing the feature pyramid network (FPN). Taking frame-by-frame images from real-time factory monitoring as the input, the feature extraction step leverages a deep convolutional layer network to identify features such as edges, corners, and basic textures. The CA attention mechanism is integrated to enhance small target features in small-scale feature maps. After feature extraction, multi-scale feature maps undergo fusion in the FPN to improve the model’s ability to detect targets of varying sizes. Finally, the model predicts bounding boxes and category confidence through a decoupled head and calculates the valve opening using rotation calculations.

#### 2.3.1. Feature Extraction

**Figure 4:**
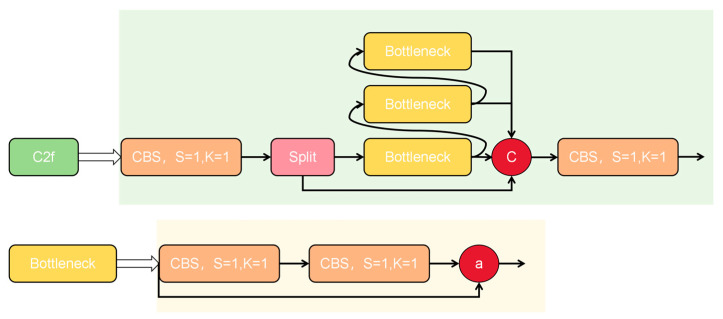
Framework of feature extraction steps based on.

The overall framework of the feature extraction step, based on regional convolutional neural networks (RCNNs), is shown in the backbone section of the YOLO framework in Figure 4. The backbone receives the input image tensor with dimensions (h_in, w_in, c_in) and processes it to extract meaningful features for object detection.

The backbone is primarily composed of convolutional layers (conv) and residual blocks. The design incorporates ideas from VGG and network-in-network architectures, enhancing feature extraction by increasing the number of channels in the network. This approach maintains the receptive field while reducing computational load, resulting in a design that is lightweight, flexible, and scalable.

The structure of the C2f residual module is illustrated in Figure 4. The main purpose of the residual link is to mitigate the gradient vanishing problem, which can occur as the number of layers increases, ensuring that the input features are effectively propagated to the deeper layers of the network. In addition to the traditional CSP structure, the C2f module includes more layer-hopping connections, removes the convolution operation in the branches, and incorporates a split operation to enrich the feature information and reduce computational effort.

The C2f module first convolves the input tensor (h_in, w_in, c_in), then splits it into two tensors (h_in, w_in, c_out). One of these tensors passes directly through n bottlenecks, while the other is concatenated with half of the output from each bottleneck layer (h_in, w_in, 0.5c_out). Finally, the residuals of (h_in, w_in, 0.5c_out) are connected. The concatenated result (h_in, w_in, 0.5 (n+2) c_out) is then output after an additional convolution operation.

#### 2.3.2. Feature Enhancement

**Figure 5:**
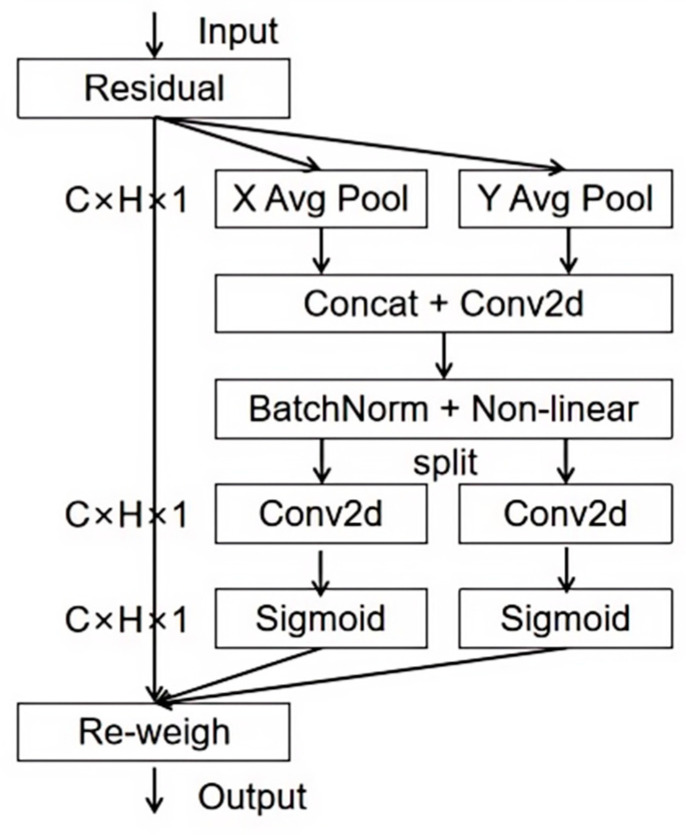
The structure of the CA module.

Small targets, like valves in the plants, often present challenges in detection due to their limited representation in high-resolution images. To address this issue, a small target feature enhancement step based on the CA mechanism is performed. Specifically, after the SPPF layer in the feature extraction stage, the CA was incorporated, which plays a crucial role in enhancing the detection performance of small targets [38]. The overall framework of the CA mechanism is shown in Figure 5.

The CA module operates by receiving input feature map tensors with dimensions (h_in, w_in, c_out). In addition to these input tensors, positional information is incorporated in the form of positional encoding vectors. These vectors are appended to the channel dimension of the input feature map tensors, effectively embedding positional information within the channel attention mechanism.

Upon embedding positional information, the CA module proceeds with the decomposition of channel attention into two distinct encoding processes. Firstly, a global encoding process aggregates features along one spatial direction, capturing global information and long-range dependencies. Secondly, a positional encoding process preserves accurate positional information along another spatial direction, ensuring the precise localization of targets.

The output of the CA module consists of enhanced feature map tensors that capture cross-channel information while simultaneously incorporating direction-aware and position-aware information. Notably, the CA module does not alter the number of channels for the original features; it merely augments the positional information. By embedding positional information within the channel attention mechanism, the CA module effectively amplifies the model’s attention towards small target features, ultimately resulting in improved detection accuracy.

#### 2.3.3. Feature Fusion and Prediction

As shown in Figure 3, the CA-enhanced feature fusion is fed into the FPN, where feature maps of different scales are fused to enable the network to capture small and large objects in the image simultaneously. This fusion approach helps the network better address the issue of scale variation in object detection tasks. Then, a decoupled head structure is used to separate classification from regression in the head section. The feature pyramid undergoes up- and down-sampling to produce feature maps at various scales, and independent detectors are then assigned to each of these feature maps. These feature maps are crucial for detecting objects of varying sizes and aspect ratios.

Then, each scale of the feature map is processed independently by detectors assigned to it. These detectors predict bounding boxes and categories. This approach effectively captures target information across different scales, enhancing the accuracy of target detection. Each detector consists of a set of convolutional and fully connected layers, with different numbers of channels being assigned to the classification branch and the regression branch, focusing on the fact that they characterize different features. Firstly, the valve type is identified through the classification task. In the regression task, the strategy used by the model is mainly based on the center approach, i.e., first finding the center/center region, then predicting the distance from the center to the four edges and regressing the bounding boxes of the valve and the coordinates of the four corners.

### 2.4. Valve Position Calculation

**Figure 6:**
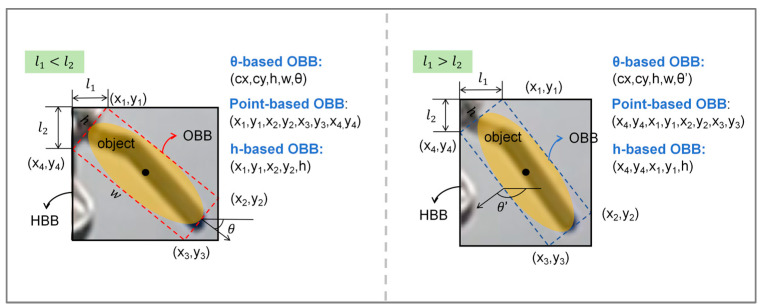
The description of the rotating frame.

After the preliminary steps, the valve frame is extracted, and then the rotation angle of the valve, i.e., the valve position, can be calculated based on the precise coordinates of the four corners of the frame. By comparing the valve position with that under normal operating conditions, if the threshold is not met, it is considered as an abnormal valve condition, and an alarm will be triggered [39]. An 8-parameter description is used for the input rotating frame based on the four vertices of the frame, i.e., x1, y1, x2, y2, x3, y3, x4, y4. However, a 5-parameter description, i.e., cx, cy, w, h, θ, is used for the loss calculation and output, as shown in Figure 6.

Calculate the angle *θ* between the rotating frame and the *x*-axis:

(1) $$\theta =\arctan\left(-\left(x_{2}-x_{1}\right),\left(y_{2}-y_{1}\right)\right)$$

The center point is calculated as:

(2) $$\left(cx,cy\right)=\left(\frac{1}{4}\sum_{i=1}^{4}x_{i},\frac{1}{4}\sum_{i=1}^{4}y_{i}\right)$$

**Figure 7:**
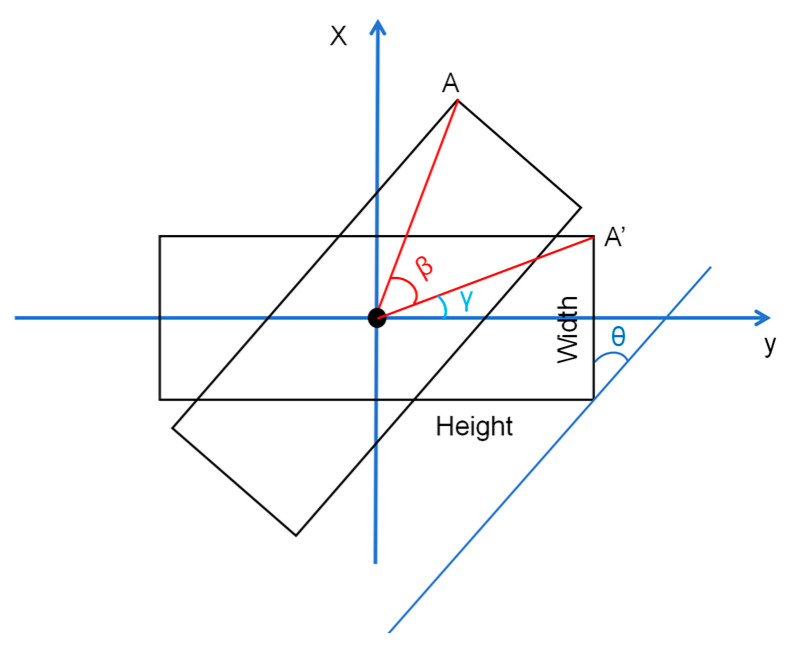
Calculation of height and width.

First, rotate the rotation box through the angle to obtain the horizontal box, and then calculate the extreme value of the left and right sides of the horizontal box to obtain the length and width value as shown in Figure 7.

To calculate the coordinates of A, as shown in the figure, *β* = 90 − *θ* is the rotation angle, and *d* is the distance from the center point to A.

(3) $$x=d\sin\left(\beta +\gamma \right)=x^{\prime }\cos\beta +y^{\prime }\sin\beta$$

(4) $$y=d\cos\left(\beta +\gamma \right)=y^{\prime }\cos\beta -x^{\prime }\sin\beta$$

The regressed rotation angle is compared with a pre-set normal threshold to monitor abnormal valve positions. In this paper, an abnormality in the valve position is defined as the rotation angle deviates from the specified correct angle range:

(5) $$\mathrm{abnormal}\;\mathrm{degree}=\left\{\begin{array}{cc} \frac{\left|\theta_{pred}-\theta_{normal\_upper}\right|}{\theta_{normal\_upper}}\times 100\% & if\;\theta_{pred}>\theta_{normal\_upper}\\ \frac{\left|\theta_{pred}-\theta_{normal\_lower}\right|}{\theta_{normal\_lower}}\times 100\% & if\;\theta_{pred}<\theta_{normal\_lower}\\ 0 & else \end{array}\right.$$

where *θ_pred_* is the valve position predicted by the regression; *θ_normal_upper_* and *θ_normal_lower_* are the upper and lower limits of the normal threshold of the valve, respectively.

## 3. Experiments

### 3.1. Experiment Design

The experiment was designed based on the industrial equipment at the practical training base of the School of Chemical Engineering at Sichuan University. To better reflect actual chemical industry production scenarios, Canon EOS 200D II camera purchased in Chengdu, China was mounted diagonally above the equipment to collect images at a speed of 25 frames per second. The captured frames were then transmitted to the monitoring model for recognition.

For each scenario, an independent camera was set up. To objectively evaluate the practical performance of this method, the following conditions of actual industrial scenarios were considered during the experimental design:

(1) The camera perspective was set diagonally above the experimental equipment, simulating an industrial surveillance scenario.
(2) The experimental scenario incorporated valves of different types, primarily with varying exterior appearances.
(3) Scenarios were designed to assess the impact of different lighting conditions on detection.
(4) Scenarios were also created to investigate the effects of valve obstruction on detection.

### 3.2. Dataset

**Figure 8:**
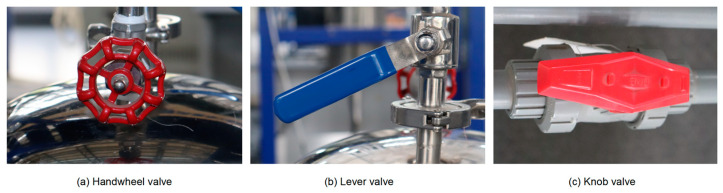
Valve categories in the experiment.

To ensure the general applicability of the method to different valves in various scenarios, experiments involving different equipment setups, valve types, and numbers of valves were designed to simulate monitoring conditions at different locations in the plants. In this experiment, three different valve categories were considered, as shown in Figure 8. Based on their appearance, they are named as handwheel valves, lever valves, and knob valves. Two sets of datasets for the experiments were obtained, as shown in Table 1. In each dataset, the images were randomly divided into training, validation, and testing sets at a ratio of 7:2:1.

YOLOv8’s efficient backbone network and versatile anchor-free detection head facilitate the feasibility of training the model on a small sample dataset. Additionally, in the industrial application context of valves, the monitored valve’s type, position, and angle of view exhibit relative consistency, rendering the currently established dataset size as adequate for supporting the training process.

**Figure 9:**
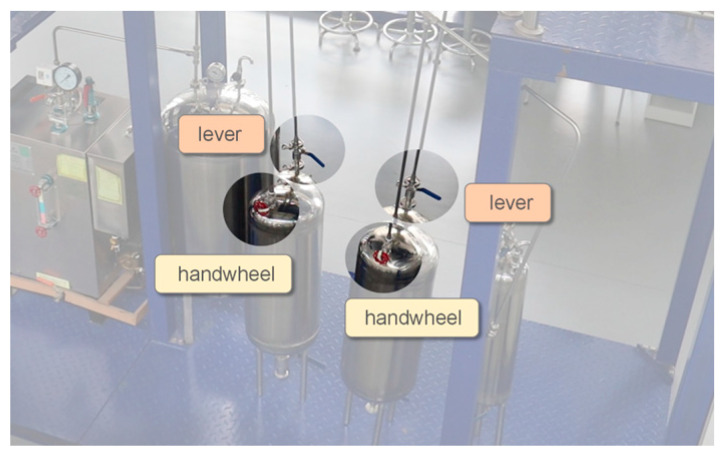
Valve designation schematic for Dataset 1.

As shown in Figure 9, the scenario of Dataset 1 mainly involves the gas phase pipeline valves of two storage tanks. It includes two handwheel valves and two lever valves on the gas phase inlet and outlet pipelines on the top of the tank.

**Figure 10:**
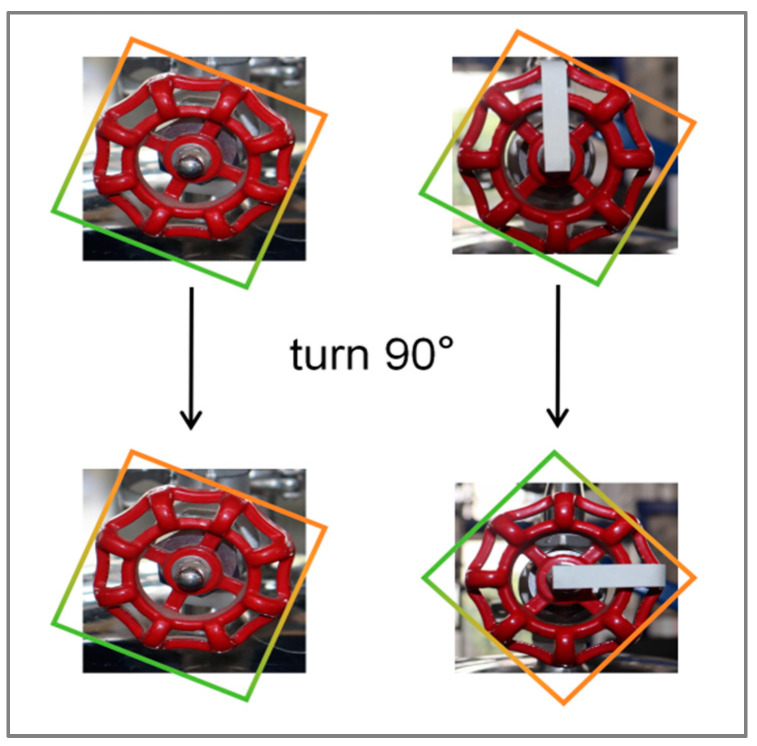
Markers on handwheel valves.

The handwheel valve has a standard circular shape and is divided into eight equal parts. Thus, every 45° of rotation makes the valve appear identical to its original position, complicating the accurate identification of the actual position. As illustrated in Figure 10, markers were added to the valve to facilitate the precise extraction of boundaries and the calculation of the rotation angle, thereby enabling an accurate determination of the valve position.

**Figure 11:**
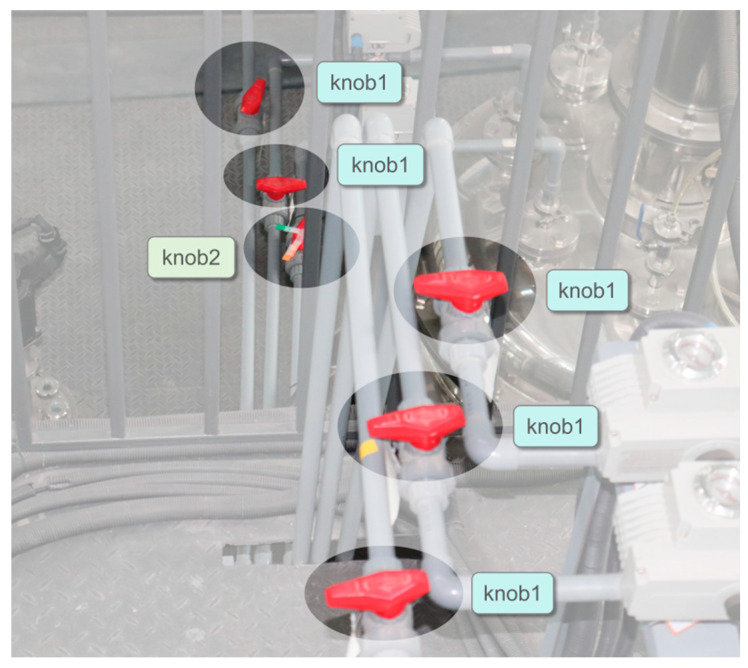
Valve designation schematic for Dataset 2.

**Figure 12:**
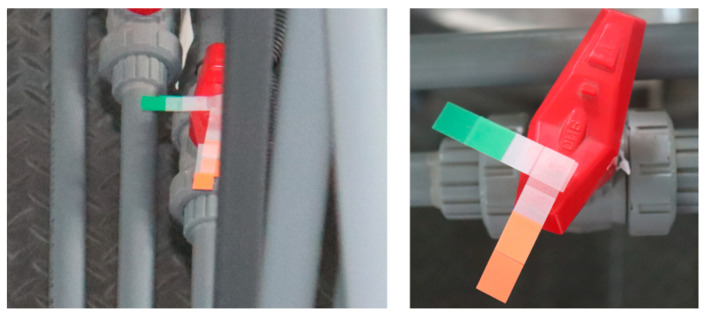
Markers on an obstructed knob valve.

As depicted in Figure 11, the scenario of Dataset 2 primarily involves six knob valves in a set of parallel pipelines. Due to the close arrangement of pipelines and the limited camera angle coverage, one of the valves is obscured. The unobstructed valve is named knob1, while the obstructed valve is named knob2. Preprocessing with markers was performed for the obstructed valve, as shown in Figure 12.

Although the training data were obtained under optimal lighting conditions, brightness adjustment was applied to some images before training. This approach allows the trained model to adapt effectively to different lighting conditions. In this study, 80 sets of test samples were established by adjusting the lights to 10–100% during nighttime in Scenario 2, aiming to test the model performance under varying lighting conditions.

Despite the initial human intervention required for valve preprocessing and brightness adjustment, this approach is more user-friendly for industrial applications compared with adding sensors or using more complex algorithms. It is characterized by its ease of operation, cost-effectiveness and does not affect the detection accuracy. The subsequent experimental results demonstrated the effectiveness of these preprocessing methods.

### 3.3. Model Training

**Figure 13:**
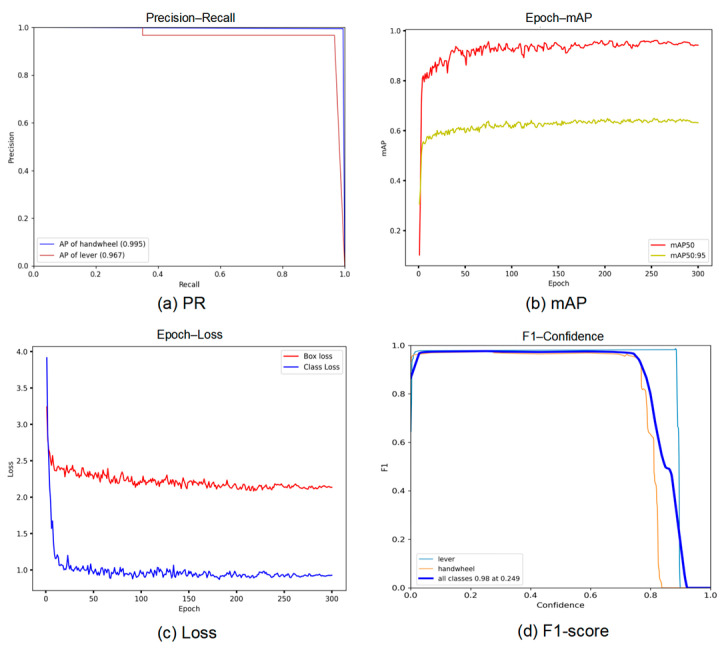
Training metrics of the proposed model.

The training environment for our model is NVIDIA RTX 4060 GPU using Python (3.8) and PyTorch (1.13.1 + cu117). Training was conducted for 300 epochs with a learning rate of 0.01. The training metrics of the model are shown in Figure 13.

Precision and recall are critical metrics for algorithm evaluation. In classification tasks, class precision is the number of true positives divided by the total number of elements predicted and labeled as positive. Recall is the number of true positives divided by the total number of elements that actually belong to the positive class. Typically, there is an inverse relationship between precision and recall, where increasing one often reduces the other. By calculating precision and recall for different thresholds, a precision–recall (P–R) curve can be plotted, showing precision as a function of recall.

(6) $$\mathrm{Precision}=\frac{\mathrm{true}\;\mathrm{positive}}{\mathrm{true}\;\mathrm{positive}+\mathrm{false}\;\mathrm{positive}}$$

(7) $$\mathrm{Recall}=\frac{\mathrm{true}\;\mathrm{positive}}{\mathrm{true}\;\mathrm{positive}+\mathrm{false}\;\mathrm{negative}}$$

The PR curve (Figure 13a) represents the precision and recall rate of the five recognized targets with an IoU threshold of 0.5. The average precision (AP) represents the area under the PR curve for each category.

(8) $$\mathrm{AP}=\int_{0}^{1}P\left(R\right)\mathrm{dR}$$

The mean average precision (mAP) denotes the average of the AP of all recognized classes. The mAP is calculated as follows:

(9) $$\mathrm{mAP}=\frac{1}{N}\sum_{i=1}^{N}{\mathrm{AP}}_{i}$$

Figure 13b shows the variation in mAP values during training. The mAP reaches above 0.85 after the 160th epoch. mAP50:95 (the average mAP for different IoU thresholds from 0.5 to 0.95) also reaches around 0.61 at the end of the iteration.

The F1-score (depicted in Figure 13d) is a crucial metric that balances accuracy and recall, providing a comprehensive evaluation of the classifier’s performance in our valve angle position detection task. Its value ranges from 0 to 1, with 1 representing a perfect classifier and 0 indicating the worst possible performance. In our dataset, the F1-score achieved is approximately 0.98, demonstrating the exceptional performance of the model.

(10) $$F1=2\times \frac{\left(Precision\times Recall\right)}{Precision+Recall}$$

All of these metrics demonstrate the effectiveness of the real-time valve knob opening monitoring scheme and show the method’s excellent performance in detecting the opening of multiple valve categories.

The BCE loss, DFL loss, and ProbIoU loss functions were weighted with certain proportions to obtain the final loss [40]. The loss stabilized after approximately 250 epochs (Figure 13c), indicating that 300 epochs were sufficient for training.

## 4. Result and Analysis

### 4.1. Model Evaluation Criteria

To objectively evaluate the detection performance of the method, the mean relative error (MRE) is introduced to analyze the detection results, as shown in the following equation:

(11) $$\mathrm{MRE}=\frac{1}{N}\sum \frac{\left|D_{pred}-D_{actual}\right|}{D_{actual}}\times 100\%$$

where *D_pred_* represents the opening angle of the valve predicted by the model, *D_actua_* represents the actual angle of the valve, and *N* is the number of testing samples. A lower MRE indicates closer alignment with the true value.

Another metric, frames per second (FPS), indicates how many frames a model can process in one second. As a general industry standard for real-time computer vision-based inspection, an average FPS of 30 frames or more is required to meet real-time requirements.

To ensure the accuracy of anomaly monitoring, the false alarm rate and the missed alarm rate were calculated. False alarms occur when rotational angles within the alarm range are not within the specified normal thresholds; missed alarms occur when the rotation angle is not within the specified normal opening thresholds but alarms are not raised. The definitions are shown below:

False alarm probability:

(12) $$P\left(\mathrm{FP}\right)=P\left(\left.\mathrm{Predict}\;\mathrm{alarm}\right|\mathrm{Actual}\;\mathrm{normal}\right)$$

False alarm probability:

(13) $$P\left(\mathrm{FN}\right)=P\left(\left.\mathrm{Predict}\;\mathrm{normal}\right|\mathrm{Actual}\;\mathrm{alarm}\right)$$

### 4.2. Results of Valves under Normal Conditions

**Figure 14:**
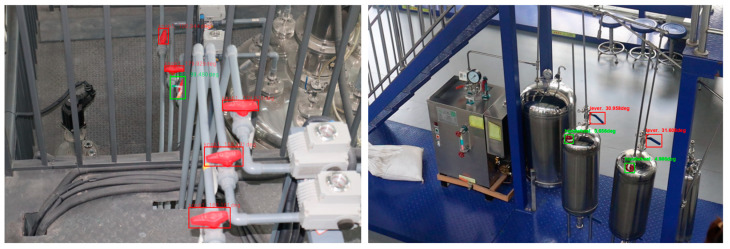
Detection results under normal conditions.

Figure 14 shows the monitoring results of the model under Scenario 1 and Scenario 2. The model accurately identifies the valve and detects the opening of the valve regardless of the scenario. When the valve position exceeds the threshold range, the color of the valve anchor box changes from green to red as an alarm indicator.

As shown in Table 2, the mean relative error (MRE) for all three types of valves is less than 12%, with an average of approximately 8%. This indicates that the method demonstrates excellent performance and a high degree of accuracy in detecting valve openness. Based on this, further determinations were performed to identify whether valves are in an abnormal state and to trigger alarms. The results show that the average false alarm rate of the model ranges from approximately 0.4% to 0.7%, and the missed alarm rate is around 0.2% to 0.8%, meeting the accuracy requirements for early warning systems in practical industrial production environments. Among the valve types, the model performs best in monitoring handwheel valves. Additionally, during the model training phase, the detection time of the proposed method is only 12.4 ms, and the FPS reaches up to 80, fully meeting the real-time requirements of industrial applications.

### 4.3. Experiment under Obscured Conditions

**Figure 15:**
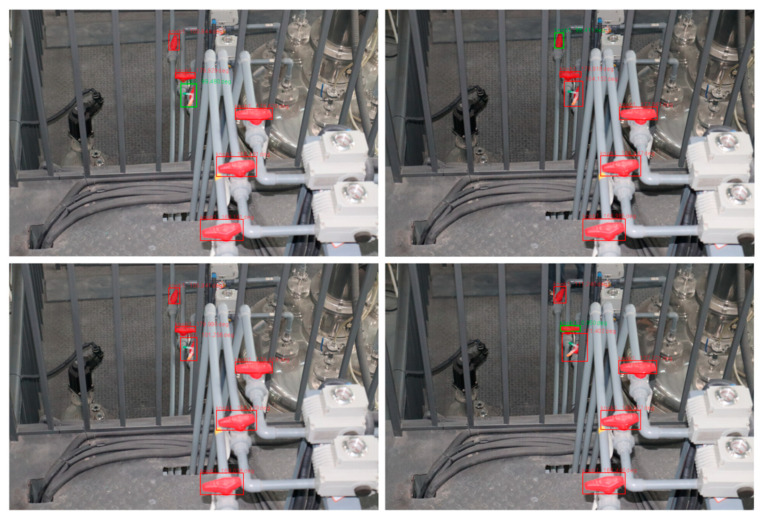
Detection results with valves obstructed.

In Scenario 2, the monitoring effectiveness of the model was tested when valves are obscured by equipment or pipes. Following the preprocessing steps outlined in Figure 12 of Section 3.2, identifiers were set for valves that were obstructed in the camera view. Ensuring that the identifiers were fully exposed to the camera, the model was then used to detect changes in the rotation of these identifiers, representing the opening size of the valve knob. These identifiers, together with the valves, are treated as specific valves, and the introduction of these identifiers does not alter the essence of the detection process. Part of the detection effect is shown in Figure 15. Because the obstructed valves and their identifiers are treated as special valves, the model successfully detected them, and the angle values fell within a reasonable range.

The experimental results, summarized in Table 3, indicate that the detection accuracy for both obstructed and unobstructed valves is above 0.98. In this scenario, the average false alarm rate and omission rate are approximately 6.00 × 10^−3^. Interestingly, the detection accuracy for obstructed valves was even higher than for regular unobstructed valves. This demonstrates that, despite the valves being obstructed, adding markers ensures that the state of the valves is clearly identified. If the features added to the markers are distinct, it can even enhance the detection effect. The results presented in Section 4.2 also suggest that the handwheel valve with an added identifier exhibits higher detection accuracy among all valve types. The preprocessing method of adding identifiers proposed in this study provides a new approach for industrial application-level detection methods.

### 4.4. Model Evaluation Criteria

**Figure 16:**
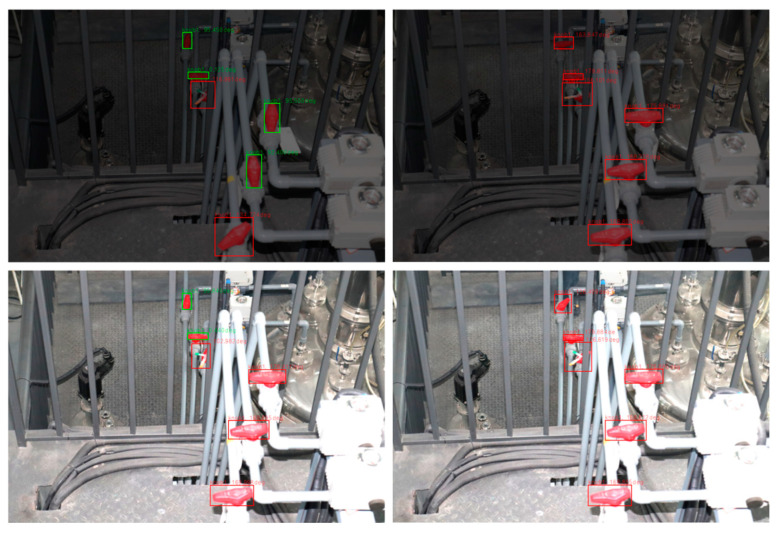
Detection results under vary lighting conditions.

The experiment regarding lighting conditions was designed based on Scenario 2. Training samples were collected under normal lighting conditions, and their brightness was randomly multiplied by 0.1–1.8 times through preprocessing for model training. The test samples were monitored under different real-world lighting intensities. As shown in Figure 16, the model can accurately identify valves and monitor valve positions under normal, excessively bright, or excessively dark conditions. Detailed results are presented in Table 4, indicating that the detection accuracy of valve positions with and without occlusion is above 0.97, and the false alarm rate and missed alarm rate are less than 1%. The findings reveal that detection accuracy considering different lighting conditions is slightly lower than under stable lighting, and the false alarm and missed alarm rates are slightly higher. This may be due to the blurring of valve boundaries and features when lighting changes are too drastic. However, the overall performance is better than models trained only on normal lighting samples without brightness preprocessing before training. This demonstrates that the proposed brightness preprocessing strategy enhances the model’s robustness and resistance to external lighting changes.

### 4.5. Results Comparison

To further elucidate the effectiveness of the proposed method, particularly the benefits of incorporating the CA mechanism, a comparative analysis was conducted with the YOLOv8n OBB model, YOLOv8x OBB model, and YOLOv9e OBB model. The results of this comparison are presented in Table 5.

As shown in Table 5, among the original YOLO series models, the YOLOv8n OBB model exhibits superior performance. The proposed model introduces the CA attention mechanism based on the original YOLOv8n OBB model framework. It demonstrates improvements in accuracy, recall, and average accuracy by 0.9%, 1.7%, and 1.1%, respectively. The introduction of the CA attention mechanism enhances the model’s ability to capture details, thereby improving its accuracy and performance while maintaining real-time processing capabilities. Certainly, the introduction of the CA module results in a higher computational cost. This is reflected in the FPS metric, where the original YOLOv8n OBB model achieves an FPS of 91, while the proposed model achieves an FPS of 80, which is 11 frames per second lower.

**Figure 17:**
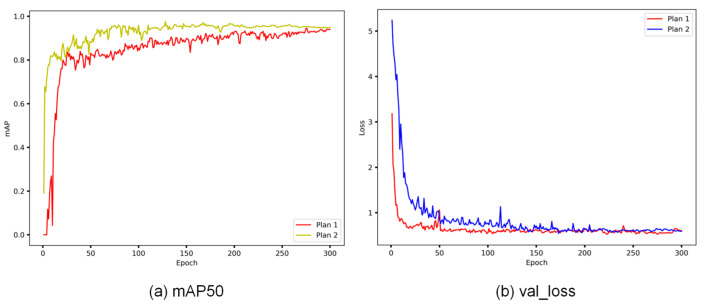
The mAP50 and val_loss of different model in the comparison experiment.

During the training process, the relationship between the mAP50 curve and the validation set loss function (val_loss) across different schemes and numbers of iterations is illustrated in Figure 17.

As shown in Figure 17, with an increasing number in training rounds, the val_loss for each scheme gradually decreases and stabilizes. Concurrently, mAP50 increases and stabilizes, indicating that the detection ability of each model improves over time. The convergence rates for the two models are similar, demonstrating that the proposed model is stable and effective.

### 4.6. Discussion and Prospects

Despite demonstrating practical efficacy and achieving excellent results, the proposed method still exhibits certain limitations. Specifically, for handwheel valves, our method can sensitively detect whether the current valve angle state is within the correct range, regardless of whether the rotation angle is less than or greater than 360 degrees. However, our research at this stage has somewhat overlooked the rotation of precisely 360-degree multiples of the correct angle range, that is, the rotation immediately after returning to the correct angle position. While the likelihood of this situation occurring after misoperation is minimal in real production processes, one potential solution is to incorporate markers that can reflect valve rotation exceeding one full revolution, such as markers linking the valve disc and the tank or valve core. We aim to address this issue more comprehensively in the next stage of our work. Currently, we utilize a camera with a fixed view angle as a visual sensor. In the future, we plan to explore the detection of varying view angles and mitigate the potential impact of differences in view angle.

## 5. Conclusions

Abnormal valve positions can significantly impact the normal operation of equipment, posing substantial risks to the stable functioning of process industry plants. This paper proposes a novel computer vision-based valve position monitoring approach. Utilizing image data collected from cameras, the method identifies and detects valve positions in real-time and issues alarms for abnormal valves. The practicality and accuracy of the proposed method have been verified through actual industrial scenarios. The main conclusions are summarized as follows:

(1) A real-time intelligent valve monitoring approach based on computer vision is introduced, incorporating the CA attention mechanism into the YOLOv8 framework to accommodate the characteristics of production scenarios.
(2) The model has been extensively tested on various types of valves, demonstrating its reliability across most prevalent valve types currently in use.
(3) Effective strategies are proposed and experimentally validated to manage scenarios involving obscured valves and varying lighting conditions.

The proposed method facilitates the low-cost, high-efficiency real-time monitoring of critical valve states in process industry plants, thereby significantly reducing operational risks and enhancing process safety management. Future integration of this method into advanced equipment, such as inspection robots, handheld terminals, and high-point cameras, is anticipated to enable more extensive and flexible industrial applications.

## Figures and Tables

**Table 1:** Datasets under normal conditions.

Set No.	Number of Valves	Valve Category	Dataset Size
1	4	2	277
2	6	1	246

**Table 2:** Model performance under normal conditions.

No.	Class	MRE	AP	P(FP)	P(FN)
1	Handwheel	6.02%	0.995	4.06 × 10^−3^	2.03 × 10^−3^
2	Lever	11.01%	0.975	6.09 × 10^−3^	8.13 × 10^−3^
3	Knob	8.21%	0.981	6.83 × 10^−3^	7.75 × 10^−3^

**Table 3:** Model performance with valves obstructed.

Class	AP	P(FP)	P(FN)
knob1	0.979	7.17 × 10^−3^	8.66 × 10^−3^
knob2	0.982	5.46 × 10^−3^	4.10 × 10^−3^

**Table 4:** Model performance under vary lighting conditions.

Class	AP	P(FP)	P(FN)
knob1	0.975	8.11 × 10^−3^	9.32 × 10^−3^
knob2	0.980	5.66 × 10^−3^	4.22 × 10^−3^

**Table 5:** Ablation experimental results.

Method	Precision	Recall	mAP50
YOLOv9e OBB model	0.925	0.956	0.967
YOLOv8x OBB model	0.929	0.976	0.944
YOLOv8n OBB model	0.966	0.972	0.968
Proposed model	0.975	0.989	0.979

## Data Availability

The data presented in this study are available on request from the corresponding authors. The data are not publicly available due to privacy restrictions.
